# AMPK Promotes Larval Metamorphosis of *Mytilus coruscus*

**DOI:** 10.3390/genes13122384

**Published:** 2022-12-16

**Authors:** Wen Zhang, Yuyi Wang, Xiaomeng Hu, Zhongsheng Zhou, Youting Zhu, Xiao Liang, Jin-Long Yang

**Affiliations:** 1 Shanghai Collaborative Innovation Center for Cultivating Elite Breeds and Green-Culture of Aquaculture animals, Shanghai 201306, China; 2 International Research Center for Marine Biosciences, Ministry of Science and Technology, Shanghai Ocean University, Shanghai 201306, China

**Keywords:** *Mytilus coruscus*, *AMPK* genes, metamorphosis, activator, inhibitor, RNA interference

## Abstract

Metamorphosis is a critical process in the transition from planktonic life to benthic life for marine invertebrates, which is accompanied by a large amount of energy consumption. Previous studies have proved that AMP-activated protein kinase (AMPK), as a vital energy regulator, plays a prominent role in mediating the growth and development of terrestrial animals. However, its function in the growth and development of marine invertebrates, especially in metamorphosis, remains elusive. This study explored the function of AMPK in the larval metamorphosis of *Mytilus coruscus*. The full-length cDNA of *AMPK* genes in *M. coruscus* was cloned and characterized, which is composed of three subunits, *McAMPKα*, *McAMPKβ*, and *McAMPKγ*. Pharmacological tests demonstrated that through the application of an AMPK activator, AMP substantially enhanced the larval metamorphosis rate (*p* < 0.05). By contrast, the larval metamorphosis rate decreased significantly after being treated with the AMPK inhibitor Compound C (*p* < 0.05). *McAMPK* gene knock-down resulted in a reduction in *McAMPK* gene expression (*p* < 0.05), and the larval metamorphosis of *M. coruscus* was significantly restrained (*p* < 0.05). These results indicated that AMPK signaling is vital in the larval metamorphosis of *M. coruscus*, which advances further understanding in exploring the molecular mechanisms in the metamorphosis of marine invertebrate larvae.

## 1. Introduction

*Mytilus coruscus* is extensively spread along the coastline of East Asia and is one of the main economical shellfish for aquaculture in China [1]. Nevertheless, the wild populations of *M. coruscus* have decreased tremendously due to human over-harvesting and marine environmental changes in recent years. Thus, most of the seedlings are raised indoors, and it is still difficult to break the technical bottleneck of the high mortality of artificial seedlings during larval metamorphosis, which restricts the sustainable development of the hard-shelled mussel breeding industry [2]. Like most marine invertebrates, *M. coruscus* undergoes a crucial process of metamorphosis to complete the transition from the planktonic stage to the benthic stage for survival. Thus, resolving the metamorphosis mechanism of *M. coruscus* larvae is conducive to the mussel aquaculture industry.

Metamorphosis means a special transformation in the life cycle of animals from larvae to juveniles, during which the morphology, physiology, and ecology of creatures will be adjusted profoundly [3]. This meaningful event occurs throughout the animal kingdom; amphibians [4], fish [5], and terrestrial and aquatic invertebrates [6,7,8] all undergo metamorphosis to survive better during their life. During the metamorphosis process, the larvae go through many drastic morphological changes; for some marine mussels, this includes the disappearance of the face plate, the shedding of the ciliary ring, the rapid growth of gill filaments, and the production of adult shells so as to develop to post-larvae and grow into adults, which need to consume a large amount of energy [9]. Some investigations have indicated that α2-adrenergic receptors [6], nitric oxide [10], adenosine kinase [11], and other signals can govern the metamorphosis process of marine mussel larvae; however, the mechanism of metamorphosis remains poorly understood.

As a master molecular sensor, AMP-activated protein kinase (AMPK) is fundamental in regulating cell energy homeostasis, organism development, and tissue growth [12,13,14]. Eukaryotes possess a highly elegant mechanism for detecting insufficient cellular ATP levels through the serine/threonine kinase AMPK compound [14], which is made up of one α catalytic subunit (α1 and α2) and two regulatory subunits, β (β1 and β2) and γ (γ1, γ2, and γ3), respectively [14,15]. When cells encounter energy stress, AMPK serves as a metabolic sensor, stimulating catabolic processes to produce additional ATP while suppressing anabolism to achieve energy balance [14,16,17]. In other words, AMPK maintains the balance of energy metabolism by regulating the synthesis and transformation of fat, protein, and glucose [18,19,20,21]. Furthermore, AMPK is in charge of maintaining germline quiescence in *Caenorhabditis elegans* larvae by changing the germline chromatin landscape to preserve germ cell integrity and regulating larvae metabolism to promote their smooth growth into adults [22]. When AMPK is activated, it enhances oxidative phosphorylation and the usage of pyruvate and allows *Drosophila melanogaster* to prune their dendrites of sensory neurons to complete developmental transitions [23].

Metamorphosis is a vital process in the life of marine invertebrates, which is accompanied by huge energy consumption. Research has shown that protein contributes the most energy to oyster larval metamorphosis, accounting for 69.3% of the total energy, followed by fat (24.3%) and sugar (6.4%) [24]. Moreover, proteins involved in glycolysis and fatty acid metabolism were upregulated during the metamorphosis of *Balanus amphitrite* [25]. This evidence indicates that energy metabolism plays a significant role in larval metamorphosis. As a fundamental energy-regulating signal, the role of AMPK in insect metamorphosis has been revealed, but the function of AMPK in the metamorphosis of marine invertebrates is rarely reported, especially in *M. coruscus*.

In this study, the full-length cDNA of *AMPK* in *M. coruscus* was cloned and characterized, which is composed of three subunits, named *McAMPKα*, *McAMPKβ*, and *McAMPKγ*. Pharmacology and RNA interference (RNAi) experiments demonstrated that when AMPK signaling was inhibited, the larval metamorphosis was restrained, whereas activating AMPK signaling promoted larval metamorphosis. This indicates that AMPK is essential for the metamorphosis of *M. coruscus* larvae. Given the significance of *M. coruscus* in the commercial bivalve breeding industry of China, this study casts the spotlight on the role of AMPK in the larval metamorphosis of *M. coruscus*, providing a new perspective for the artificial propagation of mollusks.

## 2. Materials and Methods

### 2.1. Materials

All mussel larvae were produced in Gouqi Island, Zhoushan, China (122°44′ E; 30°73′ N). The culture method of mussel larvae was modified according to previous studies [26]. After temporary rearing for 7 days under laboratory conditions (18 °C, salinity: 30‰), the larvae were collected for RNA extraction and larval metamorphosis. All extracted RNA was kept at −80 °C after being quickly frozen with liquid nitrogen for the following experiments.

### 2.2. cDNA Full-Length Cloning and Identification of Three McAMPK Subunits

The total RNA was obtained from the tested larvae utilizing an RNAiso Plus kit (TaKaRa, Shiga, Japan) following the manufacturer’s instructions. The first strand cDNA synthesis for quantitative real-time PCR (qRT-PCR) was operated with a PrimeScriptTM RT reagent kit with gDNA Eraser (Takara, Dalian, China). Both 3′ and 5′ cDNA for rapid amplification of cDNA ends (RACE) PCR were collected with a SMARTer™ RACE 5′/3′ Kit (Clontech, Mountain View, CA, USA). 5′-RACE and 3′-RACE were accomplished using specific primers that were paired with general primers, respectively (Table 1). The touchdown PCR and nested PCR of 5′-RACE and 3′-RACE and the subsequent experimental operation were performed based on the method of Zhu and Li [10,27].

### 2.3. Sequence Analysis of McAMPK Genes

The amino acid sequences were predicted utilizing ORF finder (http://www.ncbi.nlm.nih.gov/gorf/gorf.html (accessed on 14 May 2021)). Expasy (http://www.expasy.org/tools/protparam.html (accessed on 14 May 2021)) was utilized to predict the molecular weight and theoretical pI of protein. The protein domains were predicted by SMART (http://smart.embl-heidelberg.de/ (accessed on 14 May 2021)). SOPMA (https://npsa-prabi.ibcp.fr (accessed on 14 May 2021)) and SWISS-MODEL (https://www.swissmodel.expasy.org/ (accessed on 7 November 2022)) online programs were used to predict McAMPK secondary and tertiary structures, respectively. The predicted amino acid sequences of McAMPKs aligned with other organisms were performed by DNAman (Version 6). MEGA 7.0 was applied to build phylogenetic trees using the maximum likelihood method [28], and the bootstrap replicates were set to 1000 [29].

### 2.4. Pharmacologic Experiment of Larval Metamorphosis

In this study, three AMPK activators, Adenosine monophosphate (AMP for short), Metformin (MET for short), and Acadesine (AICAR for short) and one AMPK inhibitor Compound C (CC for short) were used to treat mussel larvae (Table 2). The induction tests of larval metamorphosis by AMP, MET, and AICAR were performed using the strategies outlined in earlier research, and the tested larvae were continuously immersed in a test solution of activators during the entire test [18,30].

Each group of 20 larvae was immersed in a glass Petri dish (Ø 60 mm × 19 mm height) with autoclaved filtered seawater (AFSW), and the chemical solution and the total volume was 20 mL. Each test group—Blank (AFSW), EPI (epinephrine, 10^−4^ M), AMP, MET, and AICAR—was set up with 6 replicates. Blank was set as a blank control, and EPI was a positive control. EPI-inducted tests on larvae referred to the procedures represented by Yang [38]. After being treated with the working solutions of AMPK antagonist—CC for 2 h—the pediveligers were washed in a glass dish containing AFSW to remove the surface reagent. Then, the larvae were exposed to the AMP solution continuously. The group was named CC+AMP. The preparation of chemical stock solutions and working solutions was based on previous methods [30].

### 2.5. RNAi

The small interfering RNA (siRNA) sequences designed for *McAMPKs* were all synthesized by GenePharma Co. Ltd. (Shanghai, China). The confirmation of the negative control sequence (nonsense siRNA) referred to a previous study [27]. The siRNA sequences are shown in Table 3.

The siRNA was transfected into *M. coruscus* pediveligers by electroporation using the procedures published in a previous study [27]. Briefly, about 200 pediveliger larvae were washed with AFSW before being transferred into a 1.5 mL centrifuge tube (Rnase-free, Dnase-free), which contained 1 mL AFSW (as a blank control) or 1 mL AFSW with 1.2 μg nonsense siRNA (as a negative control group) or 1 mL AFSW with 1.2 μg *AMPKα* siRNA, 1.2 μg *AMPKβ* siRNA, or 0.8 μg *AMPKγ* siRNA (as experimental groups), respectively; then, the samples were incubated for 5 min. Subsequently, the mixtures of siRNA and larvae were sucked from the centrifuge tube into the electroporation reaction cup, and the samples were electroporated with square-wave pulses using Gene Pulser Xcell (Bio-Rad, Hercules, CA, USA). Next, the electroporated mixtures of siRNA and larvae were kept at 25 °C for 10 min; then, they were transferred to the fresh AFSW for 48 h. Finally, the larvae were collected for extracting total RNA and detecting the knock-down effect of the target genes (*McAMPKα*, *McAMPKβ*, and *McAMPKγ*) by qRT-PCR. The experiment was repeated independently 4 times. The experimental groups are shown in Table 4.

### 2.6. Larval Metamorphosis and RNAi

The RNAi experiments were performed as described above. After being electroporated, the experimental groups were treated with AMP to observe the larval metamorphosis. In this test, the metamorphosis bioassay was divided into nine groups: the experimental groups with electroporation siRNA (siNC+AMP, siAMPKα+AMP, siAMPKβ+AMP, and siAMPKγ+AMP) and the control groups without siRNA (EPI, Blank, Control, AMP, and Control+AMP). Six replicates were set up for each group, and each replicate cultured 20 electroporated larvae. During this period, the metamorphosis and survival rate of larvae at 0, 24, 48, 72, and 96 h were observed and recorded under an Olympus stereoscopic microscope.

### 2.7. qRT-PCR Analysis of RNAi

For qRT-PCR, the total RNA of tested larvae was isolated and synthesized cDNA. The primer sequences designed for *McAMPK* genes and reference gene (*EF-1α*) are presented in Table 1 [6]. In qRT-PCR analysis, 3 biological repeats and 3 technical repeats were set up in each treatment group.

### 2.8. Statistical Analysis

The experimental data were pre-processed in Excel 2013 and analyzed by one-way analysis of variance (ANOVA) via SPSS 25.0. Significant differences were symbolized by different lowercase letters (*p* < 0.05).

## 3. Results

### 3.1. Characterization of McAMPK Genes Subsection

**Figure 1:**
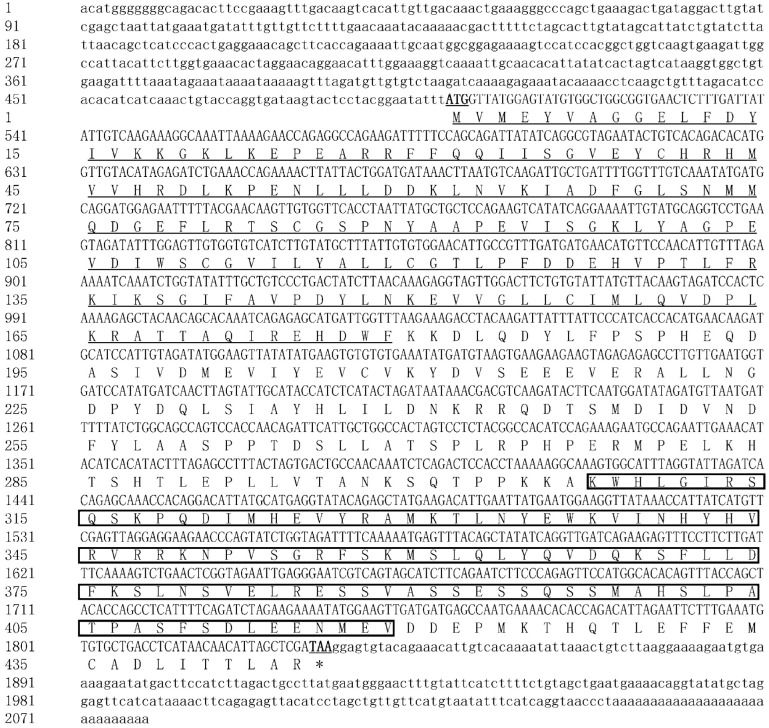
The nucleotide and predicted protein sequences of McAMPKα. The uppercase letters in cDNA represent the open reading frame (ORF), and the lowercase letters represent the 5′ and 3′ noncoding regions; * indicates stop codon. The start (ATG) and stop (TAA) codons are presented in bold and underlined. The same as below. Only underlined areas represent the S_TKc domain, and the black-framed regions represent the adenylate sensor domain.

**Figure 2:**
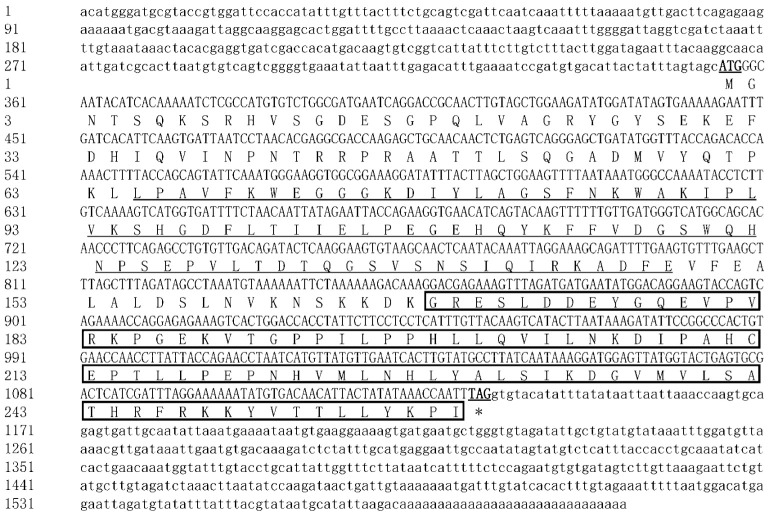
The nucleotide and predicted protein sequences of McAMPKβ. The underlined areas represent the CBM domain, and the black-framed regions represent the interaction domain, AMPKBI.

**Figure 3:**
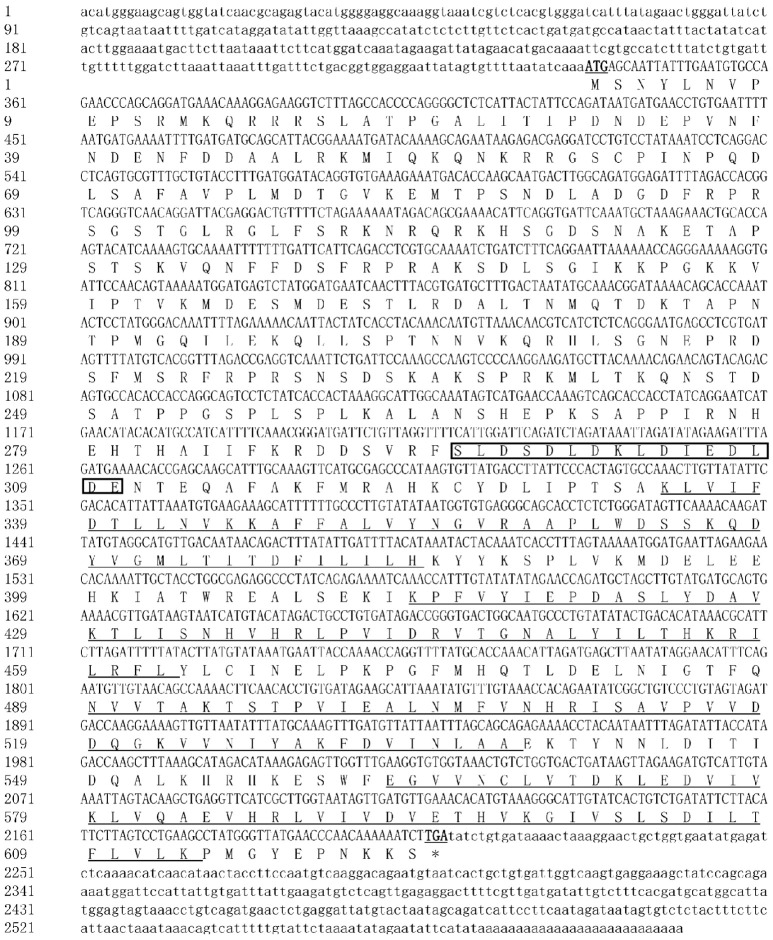
The nucleotide and predicted protein sequences of McAMPKγ. The black-framed region represents the low complexity domain, and the underlined areas represent the four CBS domain repeats.

The full-length cDNA sequences of three *McAMPK* genes—*McAMPKα*, *McAMPKβ*, and *McAMPKγ*—were cloned using RACE. The *McAMPKα* gene was 2079 bp long, which consists of a 1335 bp ORF, a 498 bp 5′-UTR, and a 246 bp 3′-UTR (GenBank accession number: ON310500). The ORF encoded 444 amino acids with a predicted molecular weight of 50.81 kDa and a theoretical pI of 5.55 (Figure 1). The SMART program results showed that McAMPKα possessed a S-TKc domain (KD), which is a catalytic domain and an adenylate sensor. The *McAMPKβ* gene was 1602 bp long, which consists of a 780 bp ORF, a 354 bp 5′-UTR, and a 468 bp 3′-UTR (GenBank accession number: ON310501). The ORF encoded 259 amino acids with a predicted molecular weight of 28.98 kDa and a theoretical pI of 7.84 (Figure 2). The SMART program results showed that McAMPKβ possessed a carbohydrate-binding module (CBM) region and AMPKBI region. The *McAMPKγ* gene was 2569 bp long, which consists of an 1872 bp ORF, a 306 bp 5′-UTR, and a 391 bp 3′-UTR (GenBank accession number: ON310502). The ORF encoded 623 amino acids with a predicted molecular weight of 70.34 kDa and a theoretical pI of 9.12 (Figure 3). The SMART program results showed that McAMPKγ possessed the conserved domains, four cystathionine β-synthase (CBS) regions. The predicted isoelectric points of the *McAMPK* genes were 5.55, 7.84, and 9.12, respectively.

**Figure 4:**
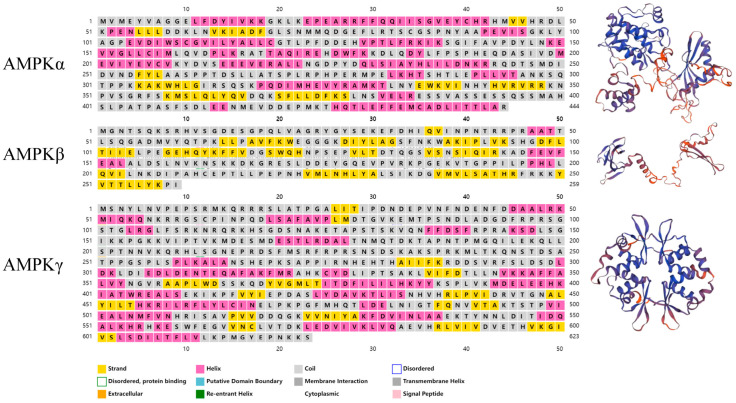
The predicted secondary and tertiary structure of McAMPK.

The predicted secondary and tertiary structures of the McAMPKα, McAMPKβ, and McAMPKγ proteins are shown in Figure 4. McAMPKα, McAMPKβ, and McAMPKγ proteins consisted of 20, 3, and 22 α helixes; 9, 16, and 16 β strands; and 29, 20, and 35 coils, respectively.

### 3.2. Multiple Sequence Alignments and Phylogenetic Analysis of McAMPKs

**Figure 5:**
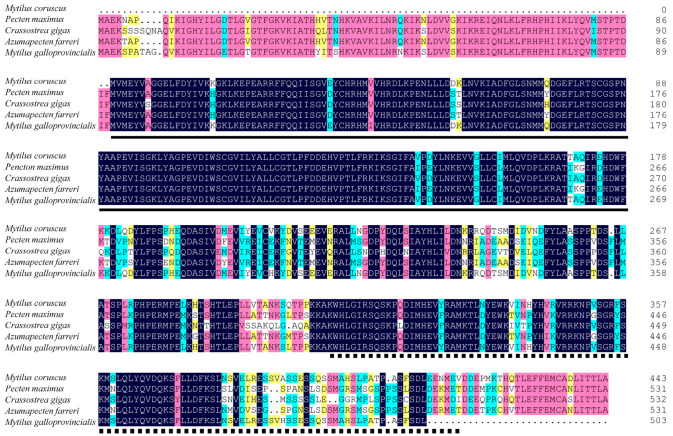
The multiple sequence alignment of McAMPKα with other mollusks. The dark blue area: complete similarity; the pink area: 75% similarity; the blue area: 50% similarity; and the yellow area: more than 33% similarity. The same as below. The underlined area represents the S_TKc domain. The square-dotted line represents the adenylate sensor domain. The accession number of the AMPKα sequence used: *Pencten maximus* (XP_033736754.1), *Crassostrea gigas* (XP_034311308.1), *Azumapecten farreri* (QFR39800.1), and *Mytilus galloprovincialis* (VDI56011.1).

The predicted McAMPKα protein shares a high sequence identity with *Mytilus galloprovincialis* (76.26% similarity), *Pecten maximus* (66.10% similarity), *Azumapecten farreri* (66.10% similarity), and *Crassostrea gigas* (64.25% similarity) (Figure 5).

**Figure 6:**
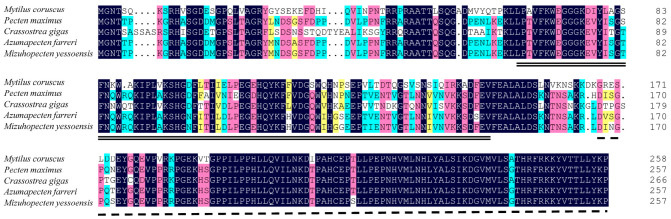
The multiple sequence alignment of McAMPKβ with other mollusks. The double underline area represents the CBM domain. The dashed line area represents the AMPKBI domain. The accession number of the AMPKβ sequence used: *Pecten maximus* (XP_033735434.1), *Crassostrea gigas* (XP_011450446.2), *Azumapecten farreri* (QFR39801.1), and *Mizuhopecten yessoensis* (XP_021357013.1).

The predicted McAMPKβ protein shares a high sequence identity with *Pecten maximus* (68.08% similarity), *Mizuhopecten yessoensis* (68.08% similarity), *Azumapecten farreri* (67.31% similarity), and *Crassostrea gigas* (67.16% similarity) (Figure 6).

**Figure 7:**
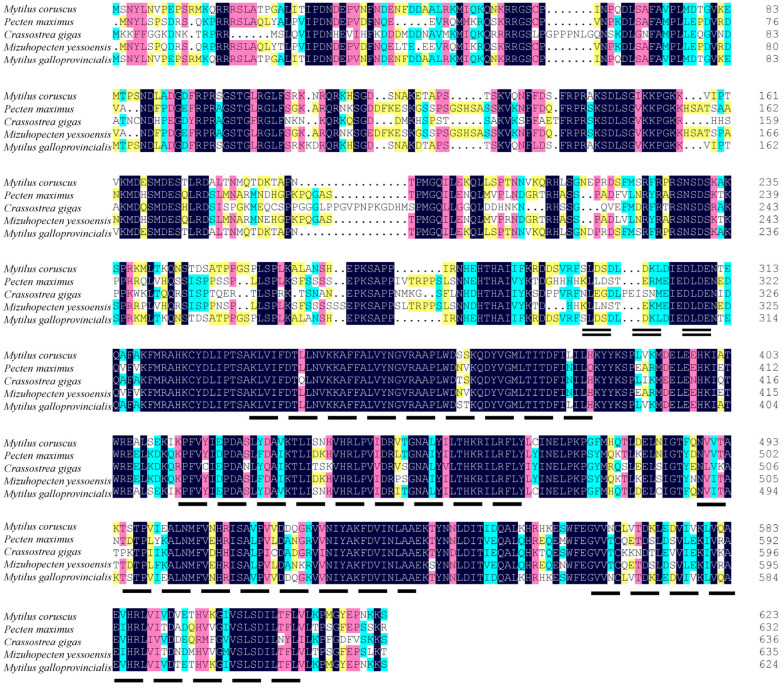
Multiple sequence alignment of McAMPKγ with other mollusks. The double-dashed line area represents the CBS domain; the long-dashed line area represents the low complexity. The accession number of the AMPKγ sequence used: *Pecten maximus* (XP_033762248.1), *Crassostrea gigas* (XP_034310765.1), *Mizuhopecten yessoensis* (XP_021369706.1), and *Mytilus galloprovincialis* (VDI78590.1).

The predicted McAMPKγ protein shares a high sequence identity with *Mytilus galloprovincialis* (98.24% similarity), *Pecten maximus* (64.55% similarity), *Mizuhopecten yessoensis* (63.48% similarity), and *Crassostrea gigas* (59.24% similarity) (Figure 7).

**Figure 8:**
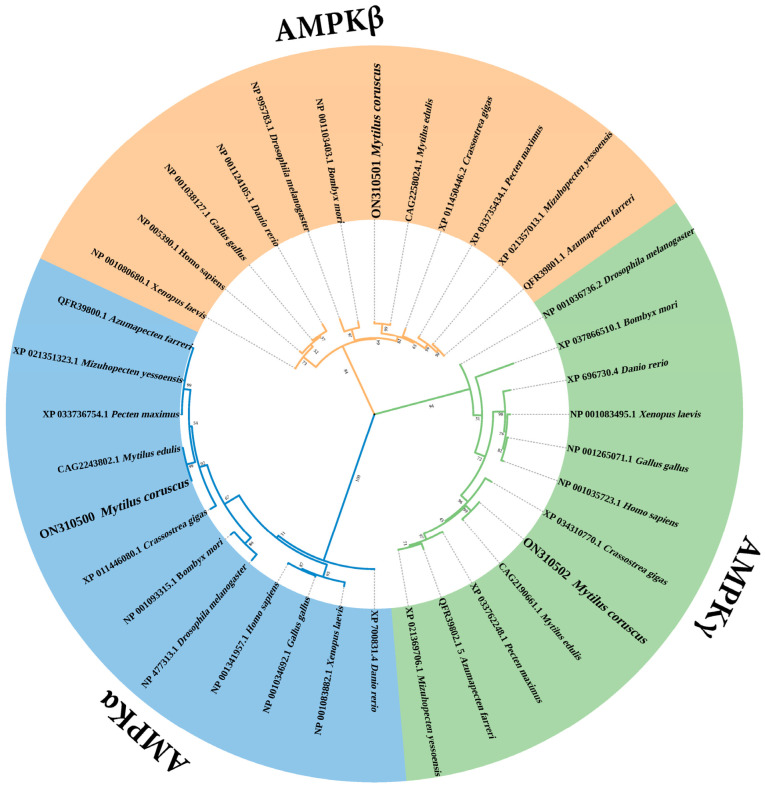
A phylogenetic analysis of McAMPKs. Numbers at the tree present the bootstrap values from 1000 replicates.

A phylogenetic tree was formed using AMPK protein sequences found in *M. coruscus*, other shellfish, insects, batrachian, and mammals (Figure 8). The phylogenetic tree presents three major clusters in accordance with the three various subunits of AMPK, which provides firm phylogenetic evidence for the identities of the *McAMPK* genes. McAMPKs have a close evolutionary relationship with other bivalves but are far away from insects and vertebrates.

### 3.3. AMPK Inhibitor and Activators Regulate M. Coruscus Larval Metamorphosis

**Figure 9:**
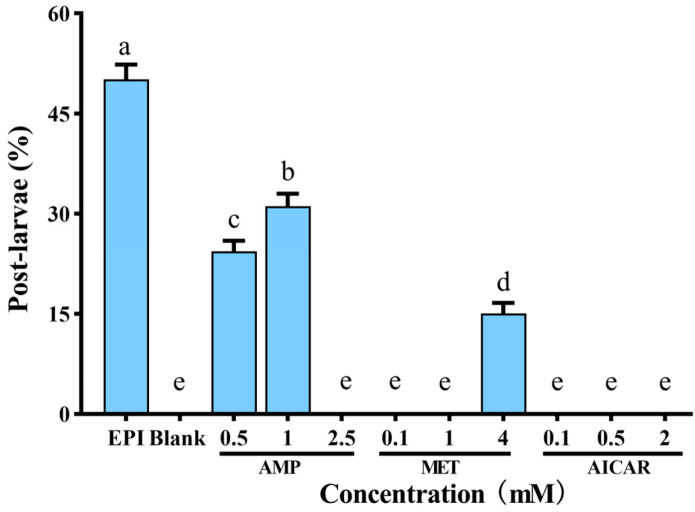
Larval metamorphosis of *M. coruscus* in response to AMPK activators at 72 h. EPI: epinephrine (10^−4^ M); Blank: autoclaved filtered seawater (AFSW); AMP: Adenosine monophosphate; MET: Metformin; and AICAR: Acadesine. The abscissa values represent the different test concentrations of the medicines.

**Figure 10:**
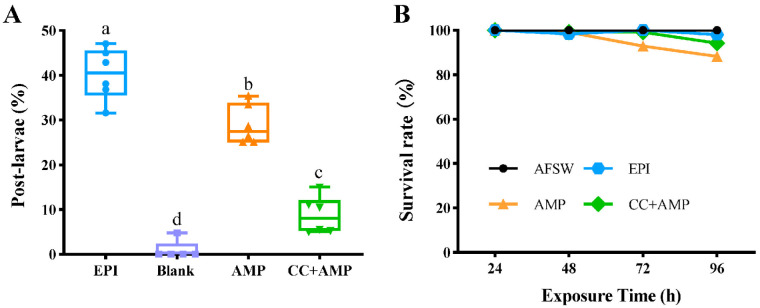
The larval metamorphosis and survival rate of *M. coruscus* at 72 h after being treated with AMPK inhibitors. (**A**) The rate of post-larvae. (**B**) The survival rate at different times.

To investigate the function of AMPK, three AMPK activators, such as AMP, MET, and AICAR, as well as the inhibitor, CC (5 × 10^−5^ mM), were utilized to test pediveligers, and the metamorphosis rate was compared with the blank control (Blank) and the positive control (EPI) (Figure 9 and Figure 10A). A total of 0.5 mM AMP had good induction for pediveligers to undergo metamorphosis, whose inductivity was significantly higher than the group Blank and the other two activators (*p* < 0.05) and had a higher survival rate (Supplementary Figure S1). The post-larvae rate of group CC+AMP treated with CC for 2 h and then treated with AMP for 72 h was significantly lower than the group AMP, which was only treated with AMP (*p* < 0.05, Figure 10A), and the larval survival rates among the groups were unaffected by the different compound treatments (*p* > 0.05, Figure 10B).

### 3.4. Validation of RNAi Effect of McAMPK Genes

**Figure 11:**
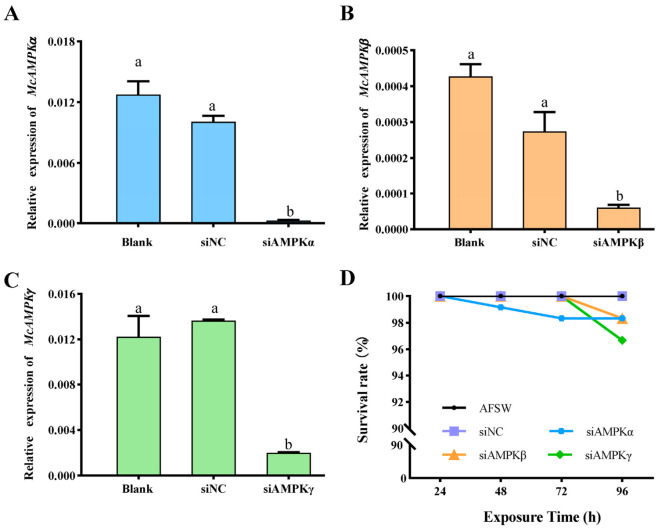
The effects of RNAi on the relative expression of *McAMPK* genes and the survival rate of *M. coruscus*. (**A**) *McAMPKα* expression in electroporated with si*AMPKα* and control larvae, (**B**) *McAMPKβ* expression in electroporated with si*AMPKβ* and control larvae, (**C**) *McAMPKγ* expression in electroporated with si*AMPKγ* and control larvae and (**D**) The survival rate of different groups electroporated with si*AMPKα*, si*AMPKβ* and si*AMPKγ*, respectively.

To detect the effect of RNAi, the gene expression of the *McAMPK*s of the electroporated larvae and control larvae were analyzed with qRT-PCR technology. The expression of the reference gene *EF-1α* was not significantly varied among the different groups (Supplementary Figure S2). It was discovered that the expression of the *McAMPKα* gene was ablated in the electroporated larvae exposed to *McAMPKα* siRNA in contrast with the blank control without siRNA (Blank) and the negative control treated with NC siRNA (siNC, *p* < 0.05, Figure 11A), as well as *McAMPKβ* and *McAMPKγ* (*p* < 0.05, Figure 11B,C). Meanwhile, after 96 h, no significant difference was found in the survival rate of larvae among the groups with siRNA transfection and the blank group (*p* > 0.05, Figure 11D). The above experimental results showed that electroporation can effectively inhibit the higher expression level of *McAMPK* genes without affecting the survival status, so it can be used for the subsequent function research of *McAMPK* genes.

### 3.5. siRNA Transfection of McAMPK Genes Inhibits M. Coruscus Larval Metamorphosis

**Figure 12:**
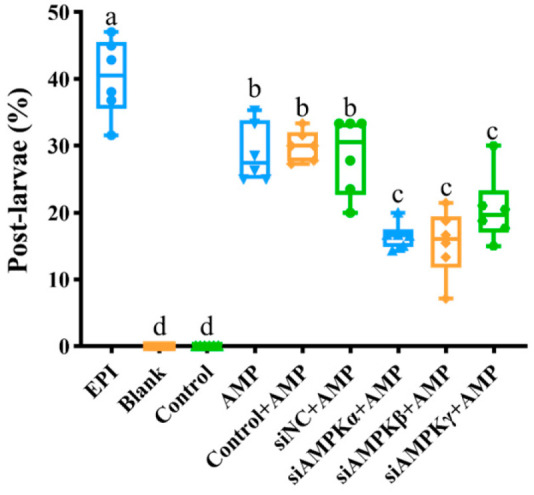
The larval metamorphosis of *M. coruscus* at 72 h after *McAMPK* RNAi. Control: electroporation; Control + AMP: electroporation + AMP; siRNA + AMP: negative control siRNA + electroporation + AMP; siAMPKα + AMP: *McAMPKα* siRNA + electroporation + AMP; siAMPKβ + AMP: *McAMPKβ* siRNA + electroporation + AMP; and siAMPKγ: *McAMPKγ* siRNA + electroporation + AMP.

In the metamorphosis assays, the pediveliger larvae only with electroporation (Control) were unable to complete metamorphosis, as well as Blank (Figure 12). The post-larvae of the pediveliger larvae that were only being treated with EPI or AMP were 42.24% ± 2.36 and 28.92% ± 1.81, respectively. The groups that were electroporated and AMP-induced (Control+AMP) and electroporated with NC siRNA and AMP-induced (siNC+AMP) were 29.99% ± 1.47 and 28.55% ± 2.36, respectively, which were slightly decreased compared with group EPI. The post-larvae of the pediveliger larvae that were treated with AMP coupled with electroporation with *McAMPKα* siRNA (siAMPKα+AMP), *McAMPKβ* (siAMPKβ+AMP), and *McAMPKγ* siRNA (siAMPKγ+AMP) significantly inhibited larval metamorphosis (*p* < 0.05, Figure 12) compared with group AMP, Control+AMP, and siNC+AMP. The above results showed that the knock-down of *McAMPK* genes by RNAi can significantly reduce the metamorphosis rate of pediveliger larvae.

## 4. Discussion

Metamorphosis is an influential process for the growth and development of many shellfish larvae, which is closely related to the survival of larvae and the economic benefits of aquaculture. In the process of metamorphosis, various physiological changes occur in the organism, which requires much energy. As a master cellular energy regulator, AMPK is essential for organism growth, development, autophagy, and metabolism [14,16]. It is well known that AMPK is essential in mammalian development, and some progress has also been made in insect development and metamorphosis [39,40,41]. Nonetheless, it is still uncertain whether AMPK serves a comparable function in marine invertebrates. In this study, the full length and molecular characteristics of the *AMPK* genes of *M. coruscus* were cloned and identified. In addition, through pharmacological and RNAi experiments, it was found that the inhibition or activation of the AMPK signal could reduce or promote the metamorphosis of larvae, respectively, which preliminarily proved that AMPK is fundamental in larval metamorphosis.

The unique *AMPKα*, *AMPKβ*, and *AMPKγ* transcript sequences were obtained by comparing the internal *M. coruscus* transcriptome data with the NCBI database. Our study cloned three AMPK subunits of *M. coruscus*, namely, *McAMPKα*, *McAMPKβ*, and *McAMPKγ*. The molecular characteristics and the potential function in the metamorphosis of *M. coruscus* were analyzed and explored. The McAMPKα protein was predicted to comprise two major conserved domains: the catalytic domain S_TKc and adenylate sensor. Furthermore, the conserved sequence serves as the core structure in AMPKα subunits and is phosphorylated by an upstream kinase (LKB1, CaMKIK) [42,43,44]. The predicted McAMPKβ protein contained a CBM domain and an interaction domain AMPKBI. The McAMPKγ protein is comprised of four CBS domains. The results of the predicted domains were consistent with *Scophthalmus maximus* [45], *Ruditapes philippinarum* [46], *Ctenopharyngodon idellus* [47], and so on. The alignments of the predicted amino acid sequences and the tertiary structures of the three subunits of McAMPKs share a high identity with other species, and the phylogenetic tree presented McAMPK as conserved cross from mollusk to vertebrates, which suggests that AMPK proteins were evolutionarily conserved [46,48].

As a molecular hub for cellular metabolic control, AMPK is essential for controlling organism growth and development [22,39,49]. For instance, AMPK could promote protein phosphatase 2A (PP2A) during *Bombyx mori* and *D. melanogaster* metamorphosis in response to high amounts of 20-hydroxyecdysone (20E), resulting in limiting growth speed and body weight [39]. When AMPK signaling is triggered, the synthesis of 20E in *Hyphantria cunea* larvae is blocked, which leads to the delay of larval metamorphosis [50]. Thus, to explore the involvement of AMPK in the metamorphosis of marine invertebrates, the inhibitor or activator of AMPK was used in the metamorphosis experiment of *M. coruscus*, and the *AMPK* genes were knocked down with siRNAs. As an upstream signal, AMP is the key signal to activate AMPK [43,51]. As a recognized inhibitor of AMPK, Compound C is widely used in AMPK studies [39,49,52], and it has been demonstrated to be efficient in inhibiting AMPK signaling in oysters [29]. In this study, the results showed that AMP can dramatically promote the metamorphosis of pediveligers, while CC can significantly decrease the induction activity of AMP on larval metamorphosis. According to our RNAi test, the decrease in *AMPK* gene expression inhibited the pediveligers to metamorphose into plantigrades. These results are consistent with previous studies [23,52,53]. This suggests that AMPK is required for the larval metamorphosis of mussels, but its specific mechanism in larval metamorphosis is still unclear. Furthermore, AMPK has been studied well in insect metamorphosis, and it has been found that AMPK is involved in regulating the energy metabolism during the metamorphosis of *D. melanogaster* and *B. mori* [39]. So, during the larval metamorphosis of marine invertebrates, whether there is a molecular mechanism similar to the AMPK signal of insects to regulate dynamic energy metabolism remains to be further studied.

## 5. Conclusions

In conclusion, three *AMPK* genes (*AMPKα*, *AMPKβ*, and *AMPKγ*) in *M. coruscus* were identified. The above findings demonstrated that AMPK plays a prominent role in the larval metamorphosis of *M. coruscus*. Our study could be an asset to elucidate the molecular mechanisms in the metamorphosis of economical marine shellfish, which can be valuable for mussel aquaculture.

## Figures and Tables

**Table 1:** The sequences of primers used in this study.

Prime Name	(5′-3′ Sequence)	Comment
ORF-*McAMPKα*-F	TCTTGGTGAAACACTAGGAACTGG	ORF
ORF-*McAMPKα*-R	TAGGTGGAGTCTGAGATTTGTTGGC	ORF
ORF-*McAMPKβ*-F	CAGGGAGCTGATATGGTTTA	ORF
ORF-*McAMPKβ*-R	GAGGAGGAAGAATAGGTGGT	ORF
ORF-*McAMPKγ*-F	CAGGGTCAACAGGATTACGAG	ORF
ORF-*McAMPKγ*-R	TTGAACTATCCCAGAGAGGTG	ORF
RACE-*McAMPKα*-R1	GGAGCAGCATAATTAGGTGAACC	5′ RACE
RACE-*McAMPKα*-R2	TCCGTAGGAGTACTTATCACCTGG	5′ RACE
RACE-*McAMPKα*-F1	ACTGCCAACAAATCTCAGACTCC	3′ RACE
RACE-*McAMPKα*-F2	TGAGGGAATCGTCAGTAGCATC	3′ RACE
RACE-*McAMPKβ*-R1	GAGGAAGAATAGGTGGTCCAGTG	5′ RACE
RACE-*McAMPKβ*-R2	TATCCTTTCCGCCACCTTCC	5′ RACE
RACE-*McAMPKβ*-F1	GCCCACTGTGAACCAACCTTATTAC	3′ RACE
RACE-*McAMPKγ*-R1	CGTAATCCTGTTGACCCTGACC	5′ RACE
RACE-*McAMPKγ*-R2	AGCAAACGCACTGAGGTCCT	5′ RACE
RACE-*McAMPKγ*-F1	ATATAATGGTGTGAGGGCAGC	3′ RACE
RACE-*McAMPKγ*-F2	ATCGGCTGTCCCTGTAGTAGA	3′ RACE
q-*McAMPKα*-F	CTACAACAGCACAAATCAGAG	qRT-PCR
q-*McAMPKα*-R	CATATGGATCACCATTCAAC	qRT-PCR
q-*McAMPKβ*-F	AGTATTCAATGGGAGGTGGCG	qRT-PCR
q-*McAMPKβ*-R	CAGGCTCTGAAGGGTTGTGCT	qRT-PCR
q-*McAMPKγ*-F	ATAATGGTGTGAGGGCAGC	qRT-PCR
q-*M*cA*MPKγ*-R	CCAGGTAGCAATTTTGTGT	qRT-PCR
*EF*-*1α*-RT-F	CACCACGAGTCTCTCCCTGA	qRT-PCR
*EF*-*1α*-RT-R	GCTGTCACCACAGACCATTCC	qRT-PCR

Note: “F” indicates forward primer, and “R” indicated reverse primer.

**Table 2:** Inducers used in the assay, and manufacturers and concentrations of stock and tested solutions.

Inducer	Function on AMPK	Manufacture and Cat. No.	Concentration (mM)	
			Stock Solution	Test Solution
AMP	Activator. Binding to AMPK γ to activate AMPK holoenzyme [31,32,33].	MCE, HY-A0181	5	0.1, 1, 2.5
Metformin	Activator. It can directly interact with AMPK by binding to AMPK γ [34].	MCE, HY-B0627	5	0.1, 1, 4
AICAR	Activator. An adenosine analogue, its function is similar to AMP [35,36].	MCE, HY-13417	5	0.1, 0.5, 2
Compound C	Inhibitor. Suppressed the phosphorylation of AMPK [36,37].	MCE, HY-13418A	1	5 × 10^−5^

**Table 3:** The sequence of siRNAs.

siRNA	siRNA Sequence (5′-3′)	Comment
siRNA-AMPKα	GCGGUGAACUCUUUGAUUATT	RNAi
siRNA-AMPKβ	GGUGGCGGAAGGAUAUUUATT	RNAi
siRNA-AMPKγ	GCAGAUCAUUCCUUCAAUATT	RNAi
siRNA-NC	UUCUCCGAACGUGUCACGUTT	RNAi

**Table 4:** The experimental groups list.

Group	siRNA (μg/mL)	Electroporation	EPI (10^−4^ M)	AMP (0.5 mM)
Blank	0	-	-	-
EPI	0	-	+	-
Control	0	+	-	-
AMP	0	-	-	+
Control+AMP	0	+	-	+
siNC+AMP	1.2	+	-	+
siAMPKα+AMP	0.8	+	-	+
siAMPKβ+AMP	0.8	+	-	+
siAMPKγ+AMP	1.2	+	-	+

## Data Availability

The original contributions presented in the study are included in the article/Supplementary Material.

## Supplementary Materials

The following supporting information can be downloaded at: https://www.mdpi.com/article/10.3390/genes13122384/s1, Figure S1: The survival rate of *M. coruscus* larvae at 72 h after being treated with AMPK activators. Figure S2: The expression of *EF-1α* in different groups.
