# Supplementary material for: AMPK Promotes Larval Metamorphosis of Mytilus coruscus
Source: Genes (Basel). 2022 Dec 16;13(12):2384. doi: 10.3390/genes13122384 (PMC9777882; doi:10.3390/genes13122384)
Supplement: Supplementary file 1 [file genes-13-02384-s001.zip › genes-2057563-supplementary.docx]

**Supplementary Figure S1.**


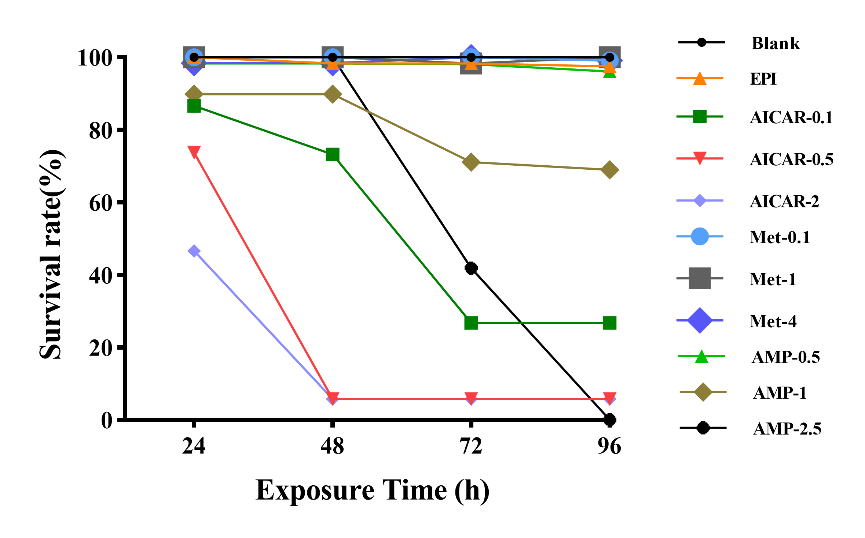


**Figure S1.** The Survival rate of *M. coruscus* larvae at 72 h after being treated with AMPK activators. The figures in the legend represent the concentration (mM).

**Supplementary Figure S2.**


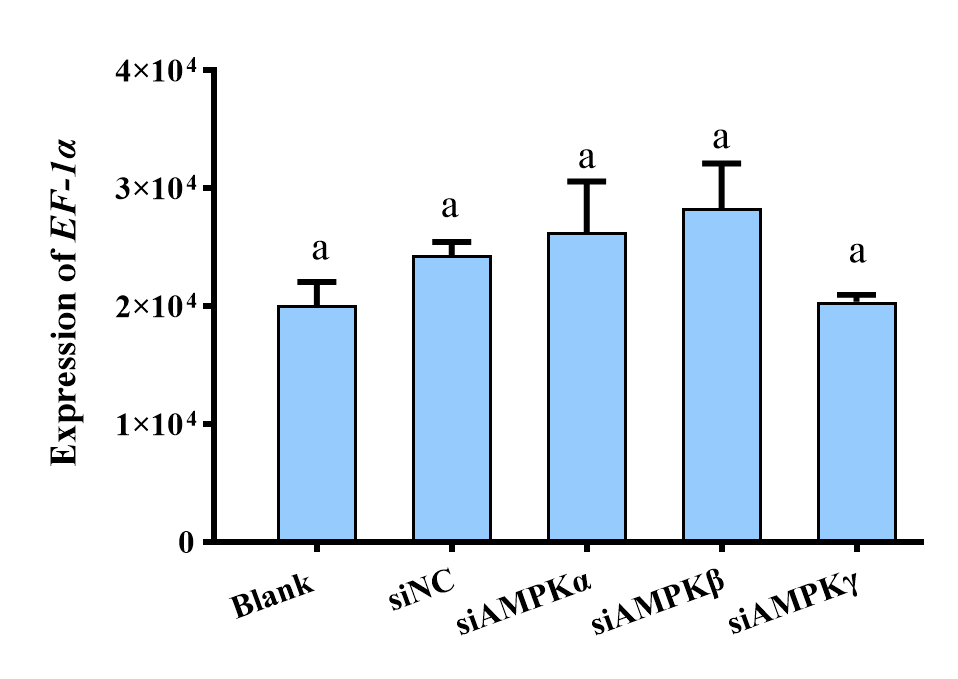


**Figure S2.** The expression of *EF-1α* in different groups.
